# Chromosome-level genome assemblies of *Musa ornata* and *Musa velutina* provide insights into pericarp dehiscence and anthocyanin biosynthesis in banana

**DOI:** 10.1093/hr/uhae079

**Published:** 2024-03-14

**Authors:** Tian-Wen Xiao, Xin Liu, Ning Fu, Tong-Jian Liu, Zheng-Feng Wang, Xue-Jun Ge, Hui-Run Huang

**Affiliations:** Key Laboratory of National Forestry and Grassland Administration on Plant Conservation and Utilization in Southern China, South China Botanical Garden, Chinese Academy of Sciences, Guangzhou 510650, China; South China National Botanical Garden, Guangzhou 510650, China; Key Laboratory of National Forestry and Grassland Administration on Plant Conservation and Utilization in Southern China, South China Botanical Garden, Chinese Academy of Sciences, Guangzhou 510650, China; South China National Botanical Garden, Guangzhou 510650, China; University of Chinese Academy of Sciences, Beijing 100049, China; Key Laboratory of National Forestry and Grassland Administration on Plant Conservation and Utilization in Southern China, South China Botanical Garden, Chinese Academy of Sciences, Guangzhou 510650, China; South China National Botanical Garden, Guangzhou 510650, China; University of Chinese Academy of Sciences, Beijing 100049, China; Key Laboratory of National Forestry and Grassland Administration on Plant Conservation and Utilization in Southern China, South China Botanical Garden, Chinese Academy of Sciences, Guangzhou 510650, China; South China National Botanical Garden, Guangzhou 510650, China; South China National Botanical Garden, Guangzhou 510650, China; Guangdong Provincial Key Laboratory of Applied Botany, South China Botanical Garden, Chinese Academy of Sciences, Guangzhou 510650, China; Key Laboratory of Vegetation Restoration and Management of Degraded Ecosystems, South China Botanical Garden, Chinese Academy of Sciences, Guangzhou, 510650, China; South China National Botanical Garden, Guangzhou 510650, China; State Key Laboratory of Plant Diversity and Specialty Crops, South China Botanical Garden, Chinese Academy of Sciences, Guangzhou 510650, China; Key Laboratory of National Forestry and Grassland Administration on Plant Conservation and Utilization in Southern China, South China Botanical Garden, Chinese Academy of Sciences, Guangzhou 510650, China; South China National Botanical Garden, Guangzhou 510650, China

## Abstract

*Musa ornata* and *Musa velutina* are members of the Musaceae family and are indigenous to the South and Southeast Asia. They are very popular in the horticultural market, but the lack of genomic sequencing data and genetic studies has hampered efforts to improve their ornamental value. In this study, we generated the first chromosome-level genome assemblies for both species by utilizing Oxford Nanopore long reads and Hi-C reads. The genomes of *M. ornata* and *M. velutina* were assembled into 11 pseudochromosomes with genome sizes of 427.85 Mb and 478.10 Mb, respectively. Repetitive sequences comprised 46.70% and 50.91% of the total genomes for *M. ornata* and *M. velutina*, respectively. Differentially expressed gene (DEG) and Gene Ontology (GO) enrichment analyses indicated that upregulated genes in the mature pericarps of *M. velutina* were mainly associated with the saccharide metabolic processes, particularly at the cell wall and extracellular region. Furthermore, we identified polygalacturonase (PG) genes that exhibited higher expression level in mature pericarps of *M. velutina* compared to other tissues, potentially being accountable for pericarp dehiscence. This study also identified genes associated with anthocyanin biosynthesis pathway. Taken together, the chromosomal-level genome assemblies of *M. ornata* and *M. velutina* provide valuable insights into the mechanism of pericarp dehiscence and anthocyanin biosynthesis in banana, which will significantly contribute to future genetic and molecular breeding efforts.

## Introduction

Banana *(Musa* spp.) comprises approximately 70 herbaceous species, which are distributed in tropical and subtropical regions of Asia and Oceania [1]. This genus is renowned for being one of the most important food crops globally. Current banana cultivars are descendants of pure *Musa acuminata* or hybrids of *M. acuminata* and several other *Musa* species [2], but ancestors of some cultivated bananas are still missing [3]. In addition, *Musa* species include many important ornamental species, such as *Musa laterita*, *Musa ornata*, *Musa rosea*, *Musa rubra* and *Musa velutina* [4, 5]. With the rapid development of third-generation sequencing technology, an increasing number of high-quality plant genomes have been assembled and released, which can facilitate crop domestication [6–9], and the advancement of ornamental plants [10–13]. Despite the significance of producing high-quality genomes, there is still a lack of genomic resources for banana cultivars, their wild relatives and ornamental species of *Musa* [but see 14, 15–22].

**Figure 1:**
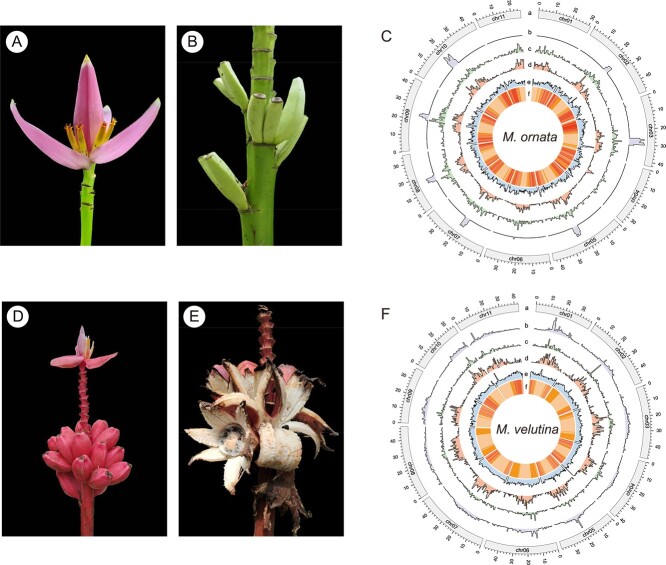
**A** and **B** Flowers and fruits of *Musa ornata*. **D** and **E** Flowers and fruits of *Musa velutina*. **C** and **F** Chromosome characterization of the Mo and Mv genome assemblies, respectively. The tracks from the outer to the inner (a–f) represent the chromosome, tandem repeat density, gypsy element density, copia element density, GC content, and gene density, respectively. These metrics were calculated in 700 kb windows.

*M. ornata* W. Roxburgh (Mo) and *M. velutina* H. Wendl. & Drude (Mv) belong to the section *Musa* of the Musaceae family and are closely related to *M. acuminata* [23]. Mo, also referred to as the flowering banana or ornamental banana, is native to Bangladesh, Myanmar and northeast India and is widely cultivated in the tropical countries. It can be identified by its pale lilac-purple bracts with small yellow apices, green peduncles and erect inflorescence (Fig. 1A and B) [24]. Mv, commonly known as the pink banana, is native to Myanmar and northeast India and is cultivated in the tropical countries. It can be easily distinguished by its brightly colored pink and hairy fruits that self-peel when mature (Fig. 1D and E) [24]. Both Mo and Mv have received the Award of Garden Merit from the Royal Horticultural Society of the United Kingdom. In addition to their ornamental value, their fruits are also a source of food for the local people [25]. These attributes make Mo and Mv desirable candidates for generating high quality genomes to aid future molecular breeding endeavors.

Self-peeling (or pericarp-dehiscent) fruits of ornamental plants have the potential to attract more animals than non-self-peeling fruits do, which can be advantageous for seed dispersal in the wild [26, 27] but may cause issues for gardeners. Pericarp dehiscence has been suggested to be correlated with degeneration of the middle lamella [28], which is the outermost layer of cell wall and is rich in pectic polysaccharides [29]. Among many biological functions, the middle lamella plays a crucial role in maintaining the structural integrity of plant tissues and organs by gluing cells together and preventing them from sliding against each other [29]. Polygalacturonase (PG; a pectinase) genes encode enzymes that degrade pectin in plant cell walls by catalyzing the hydrolysis of α-(1–4) glycosidic bonds in polygalacturonic acid chains, which produces galacturonic acid monomers and oligosaccharides as degradation products [30]. This process is intimately linked to anther dehiscence [31], fruit ripening and cracking [32], and the shedding of leaves, flowers, and fruits [33, 34]. For example, the overexpression of PG genes promotes cell separation in siliques of *Arabidopsis* and results in pericarp dehiscence [35]. Moreover, the cellulose is the major structural component of the plant cell wall, particularly the primary and secondary cell walls [36]. The cellulase (CEL) genes encode enzymes that degrade cellulose, and they are upregulated during fruit abscission in many plant species [37]. Pericarp dehiscence has been reported in Mv, *Musa schizocarpa* and some cultivars of *M. acuminata* [4, 38]. However, despite the importance of Mv as an ornamental plant and a close relative of *M. acuminata*, the molecular mechanism of pericarp dehiscence has not been investigated.

Anthocyanins are phenolic compounds that contribute to plant coloration and have important biological functions, including antibacterial effects, removal of excess reactive oxygen species, and attraction to animals for pollination [39, 40]. Anthocyanins are synthesized via the phenylpropanoid pathway, which is catalyzed by structural genes such as chalcone synthase (CHS), chalcone isomerase (CHI), flavanone 3-hydroxylase (F3H), dihydroflavonol 4-reductase (DFR), anthocyanidin synthase (ANS), and flavonoid 3-glucosyl transferase (3-GT) [41]. Previous studies have conducted comparative analyses and have indicated that anthocyanins play an important role in the formation of the purple peel of *Musa itinerans* [42], as well as the red peel of *Musa* AAA Red green [43]. Although colored bracts and fruits have great ornamental value, the regulation of the anthocyanin biosynthesis pathway in Mo and Mv remains elusive.

To provide additional genomic resources for wild *Musa* species and to explore the molecular mechanism underlying pericarp dehiscence and anthocyanin biosynthesis, we present here the chromosome-scale assemblies of Mo and Mv. These two genomes were constructed using a combination of Nanopore long-read sequencing and Hi-C scaffolding. Based on genome evolution analyses, we found that Mo and Mv had no species-specific whole genome duplication (WGD) events. The comparative analysis indicated that genome structures were relatively conserved among the two genomes and *M. acuminata*. Differentially expressed gene (DEG) analysis indicated that the upregulated genes in the mature pericarp were involved primarily in saccharide metabolic processes. Furthermore, we identified anthocyanin synthesis-related genes and PG genes that may be responsible for pericarp dehiscence. Our study lays the foundation for genetic analyses of Mo and Mv, provides insights into their genomic features, and provides solid groundwork for future endeavors aimed at crop and ornamental plant improvement.

## Results

### Genome sequencing and assembly

The genomes of Mo and Mv were sequenced and assembled in this study. In total, 34.01 Gb and 39.02 Gb of short clean Illumina reads of Mo and Mv were obtained for the genomic survey, respectively (Table S1, see online supplementary material). According to the 21-mer analysis of the Illumina reads, the haploid genome size of Mo was estimated to be 432.26 Mb, the heterozygosity was 0.37%, and the repeat content was 40.40% (Fig. S1A, see online supplementary material). The haploid genome size of Mv was estimated to be 464.33 Mb, the heterozygosity was 0.09%, and the repeat content was 41.10% (Fig. S1B, see online supplementary material). A total of 57.48 Gb and 56.34 Gb of Nanopore long reads were generated for Mo and Mv, with median read lengths of 18.46 kb and 20.29 kb, and read N50 lengths of 30.57 kb and 30.72 kb, respectively (Table S1, see online supplementary material). The Nanopore long reads were used for genome assembly. The draft genome size of Mo was 538.58 Mb, consisting of 259 contigs and a contig N50 length of 12.88 Mb. For Mv, the genome size was 498.22 Mb, comprising of 108 contigs with a contig N50 length of 18.18 Mb. The redundant sequences of the draft genomes were then removed, and the genome assemblies were polished using Nanopore and Illumina reads. Subsequently, 235.16 Gb and 271.49 Gb of clean Hi-C reads of Mo and Mv were used for scaffolding, respectively (Table S1, see online supplementary material). Thereafter, the genome assembly of Mo covered a total of 477.18 Mb and consisted of 36 scaffolds with a scaffold N50 length of 38.31 Mb. In addition, 89.66% of the sequences were anchored to 11 pseudochromosomes, with a cumulative length of 427.85 Mb (Table 1; Table S2, see online supplementary material). The genome assembly of Mv was 496.23 Mb in length and consisted of 34 scaffolds with a scaffold N50 length of 42.77 Mb; in addition, 96.36% of the sequences (478.10 Mb) were anchored to 11 pseudochromosomes (Table 1; Table S2, see online supplementary material). The GC ratios of Mo ranged from 35.23% to 46.50%, with an average of 38.62% (Fig. 1C), and Mv ranged from 32.15% to 51.22%, with an average of 38.57% (Fig. 1F). Initially, there were 38 and 37 gaps in the genomes of Mo and Mv, respectively (Table S2, see online supplementary material). After gap-closing, Mo had three gaps, two on chr01 and one on chr03, whereas Mv had six gaps, one on chr02, one on chr07, and four on chr09 (Fig. S2; Table S2, see online supplementary material). Nine and eight telomeres were identified in the Mo and Mv genome assemblies, respectively (Fig. S2, see online supplementary material), with the telomere repeat monomers of Mo and Mv being ‘AGGCCC’ and ‘AAACCCT’, respectively. The telomeric repeat numbers of Mo ranged from 110 (chr08 right end) to 4571 (chr05 right end), and those of Mv ranged from 287 (chr06 right end) to 4277 (chr01 right end) (Table S3, see online supplementary material). According to the results of the centromere statistical analysis, the lengths of the potential centromere tandem repeats (TRs) of Mo ranged from 2917 bp (chr04) to 3 841 662 bp (chr10), and those of Mv ranged from 10 177 bp (chr03) to 742 822 bp (chr02) (Fig. 1C and 1F; Table S4, see online supplementary material). The location of potential centromeric region was shown in Fig. S2 (see online supplementary material).

**Table 1:** Summary of the genome assemblies of *Musa ornata* and *Musa velutina*.

**Genome features**	***M. ornata***	***M. velutina***
Estimated genome size (Mb)	432.26	464.33
Chromosome number	2n = 2 × 11	2n = 2 × 11
Initial genome assembly size (Mb)	538.58	498.22
Contig number	259	108
Contig N50 (Mb)	12.88	18.18
Genome size after scaffolding (Mb)	477.18	496.23
Scaffold number	36	34
Scaffold N50 (Mb)	38.31	42.77
Pseudochromosome length (Mb)	427.85	478.10
Gap numbers	3	6
Telomeres identified	9	8
BUSCO assessment	98.08%	98.51%
LAI	13.68	16.81

The Mo and Mv genomes had high completeness (98.08% and 98.51%, respectively) according to the BUSCO analyses (Fig. S3, see online supplementary material). A total of 95.55% and 94.29% of the Illumina reads and 97.55% and 98.28% of the RNA reads were mapped to the genomes of Mo and Mv, respectively. The LAIs of Mo and Mv were 13.68 and 16.81, respectively. The Hi-C heatmaps showed that the pseudochromosomes of Mo and Mv were well connected along the diagonal (Fig. S4, see online supplementary material). Thus, two high-quality chromosome-scale genomes of Mo and Mv were assembled.

### Genome annotation

According to the EDTA analysis, 46.70% of the Mo genome was identified as repetitive sequences. Among the major types of TEs identified, long terminal repeats (LTRs) comprised the highest proportion and accounted for 38.97% of the genome; these included 25.86% of *Copia* and 9.76% of *Gypsy* (Table S5, see online supplementary material). For Mv, ~243 Mb (50.91%) of repetitive sequences were identified, among which LTRs were the major repeats and accounted for 42.75% of the genome. The predominant LTR was *Copia* (30.09%), followed by *Gypsy* (7.18%) (Table S5, see online supplementary material).

To identify the genes in the Mo and Mv genomes, a combination of *de novo*, transcriptome, and homolog-based annotation approaches was applied. Using protein sequences from *Ensete glaucum*, *M. acuminata*, *Musa balbisiana*, *M. itinerans*, and *M. schizocarpa* (Table S6, see online supplementary material) as a homologous database and transcriptome data from leaves, bracts, and tepals (Table S1, see online supplementary material), a total of 39 177 genes encoding 43 848 proteins were predicted with an average gene length of 4151.19 bp for the Mo genome. Among the protein-coding genes, 35 868 (91.55%) were functionally identified by the EggNOG database, with 27 428 (70.01%), 32 839 (83.82%), and 28 436 (72.58%) of the genes identified by GO, InterProScan, and Pfam, respectively (Table S7, see online supplementary material). Using the same protein sequences from five species as a homologous database and transcriptome data from leaves, bracts, tepals, pericarps, and sarcocarps (Table S1, see online supplementary material), the Mv genome was found to contain 31 256 genes encoding 36 066 proteins with an average gene length of 4800.97 bp. Of these protein-coding genes, 31 084 (99.45%) could be identified in the EggNOG database, with 21 768 (69.64%), 25 763 (82.43%), and 25 005 (80.00%) of the genes identified by GO, InterProScan, and Pfam, respectively (Table S7, see online supplementary material). In addition to protein-coding genes, 781 and 990 tRNA genes were annotated in the genomes of Mo and Mv, respectively. According to the BUSCO assessment, the protein sequences of Mo and Mv had completeness score of 95.66% and 87.67%, respectively (Fig. S5, see online supplementary material).

### Phylogeny and gene family expansion and contraction

**Figure 2:**
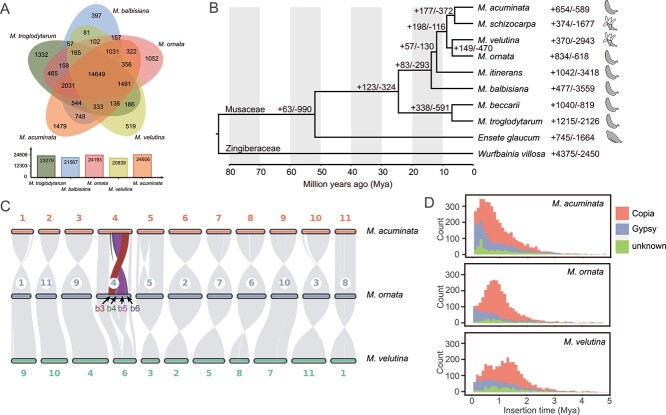
Comparative analysis of gene families between the genomes of Mo, Mv, and other species. **A** The shared and unique gene families among the five genomes of *Musa*. **B** Divergence time of 10 species based on 2641 single-copy nuclear genes. The numbers near nodes and species names indicate gene families that have expanded (+) or contracted (−). The fruit sketches indicate the dehiscence or indehiscence of pericarps when mature. **C** Genome synteny plot. b3, b4, b5, and b6 indicate large blocks with structural variation. **D** LTR insertion time (bin width = 0.1).

Protein sequences from Mo, Mv, *M. acuminata*, *M. balbisiana*, *Musa beccarii*, *M. itinerans*, *M. schizocarpa*, *Musa troglodytarum*, *E. glaucum* (Musaceae) and *Wurfbainia villosa* (Zingiberaceae; Table S6, see online supplementary material) were clustered and grouped into 34 473 gene families. We identified 24 193 gene families in the Mo genome, which was more than those characterized in the genomes of Mv (20839), *M. balbisiana* (21567) and *M. troglodytarum* (23279) but slightly less than the number of gene families in the *M. acuminata* genome (24606) (Fig. 2A). Moreover, 14 649 gene families were shared by the five *Musa* species, whereas there were 1052 unique gene families in Mo, which was greater than that in Mv (519) and *M. balbisiana* (397) but less than that in *M. acuminata* (1479) and *M. troglodytarum* (1332) (Fig. 2A). We then performed GO enrichment analysis for the unique gene families of Mo and Mv, respectively. The results showed that the unique gene families in Mo were the most significantly enriched in the GO terms ‘sulfotransferase activity’ and ‘transferase activity, transferring sulphur-containing groups’ (Fig. S6A, see online supplementary material), while the unique gene families in Mv were significantly enriched in the GO terms ‘disaccharide metabolic process’ and ‘oligosaccharide metabolic process’ (Fig. S6B, see online supplementary material).

To explore the evolutionary relationships of Mo and Mv, we identified 2641 single-copy gene families among the 10 species and used these genes for phylogenetic tree reconstruction and divergence time estimation. Our analysis showed that Mo and Mv were sisters and had close relationship with *M. acuminata* (Fig. 2B). Mo and Mv diverged at 6.87 Mya, and they diverged from *M. acuminata* at 8.67 Mya (Fig. 2B; Fig. S7, see online supplementary material). The insertion of *Copia* in *M. acuminata* and Mo peaked at 0.4 Mya and 0.8 Mya, respectively, while Mv peaked at 1.3 Mya, with a second peak occurring at 0.5 Mya (Fig. 2D).

According to the results of gene family expansion and contraction analysis, 834 and 618 gene families of Mo experienced expansion and contraction, respectively (Fig. 2B). Among the expanded gene families, 420 were significant, consisting of 1717 genes; 181 gene families were significantly contracted with 184 genes (Table S8, see online supplementary material). For Mv, 370 and 2943 gene families experienced expansion and contraction, respectively (Fig. 2B). Among the expanded gene families, 124 were significant, consisting of 565 genes; 709 gene families were significantly contracted with 680 genes (Table S8, see online supplementary material). GO enrichment analysis of the significantly expanded gene families indicated that the genes in Mo were enriched mainly in the GO terms ‘structural molecule activity’, ‘structural constituent of ribosome’, and ‘actin binding’. (Fig. S8A, see online supplementary material), while the Mv expanded genes were enriched in the GO terms ‘GTPase activity’, ‘monooxygenase activity’, and ‘chromatin’ (Fig. S8B, see online supplementary material). In contrast, the GO enrichment analysis of the significantly contracted gene families indicated that the genes in Mo were enriched mainly in the GO terms ‘ATPase-coupled transmembrane transporter activity’ and ‘primary active transmembrane transporter activity’ (Fig. S8C, see online supplementary material), while the Mv contracted genes were enriched in the GO terms ‘structural molecule activity’ and ‘GTP binding’ (Fig. S8D, see online supplementary material).

### Genome synteny, duplications, and whole genome comparisons

A synteny plot showed that the structure of most homologous chromosomes was relatively conserved among Mo, Mv, and *M. acuminata*, but inversions and translocations were observed in chr04 of Mo when comparing to Mv and *M. acuminata* (Fig. 2C). Chr04 of Mo can be divided into seven large blocks, among which block 3 was translocated with a size of 6.4 Mb and blocks 4, 5, and 6 were inversed with sizes of 1.6, 9.3, and 2.2 Mb, respectively (Fig. 2C). To ensure that these structural variations were not caused by incorrect assembly, we analysed the Hi-C signals in the surrounding regions by mapping Hi-C reads to the genome of Mo. Our analysis confirmed the presence of these variations (Fig. S9, see online supplementary material). Based on the modes of duplication, the Mo genes were classified into WGD, transposed duplication (TRD), tandem duplication (TD), proximal duplication (PD), and dispersed duplication (DD), containing 12 720, 1542, 983, 2146, and 47 164 gene pairs, respectively, and Mv into 8530, 1355, 941, 1449, and 40 033 gene pairs, respectively (Fig. S10; Table S9, see online supplementary material). After assigning a unique mode of duplication for each gene, 16 245, 1552, 1381, 1031, and 9195 unique genes of Mo were identified as WGD, TRD, TD, PD and DD, respectively, with 11 860, 1537, 1360, 677, and 8808 unique genes for Mv (Fig. S10; Table S9, see online supplementary material). The distribution map of synonymous nucleotide substitutions (Ks) (Fig. S11, see online supplementary material), together with the genome synteny plot showing a collinear pattern of 1:1 (Fig. 2C), suggested that Mo and Mv experienced at least two WGD events. We used *M. acuminata* as a reference to calculate the Ka/Ks of Mo and Mv, and our analysis showed that the two species had similar Ka/Ks distribution patterns (Fig. S12, see online supplementary material). Furthermore, we selected genes under positive selection (Ka/Ks > 1) for GO enrichment analysis, and the results showed that Mo and Mv were primarily enriched in the GO terms such as ‘plastid organization’, ‘chloroplast organization’, and ‘defense response’ (Fig. S13, see online supplementary material), suggesting that the two species may have undergone similar selective pressure. The PSMC trajectory showed that Mo and *M. acumianta* had large historical effective population sizes, which began to decrease from ~60 000 years ago to the present. In contrast to those of Mo and *M. acuminata*, Mv had a relatively small historical effective population size (Fig. S14, see online supplementary material).

### Pericarp dehiscence-related genes of mv

**Figure 3:**
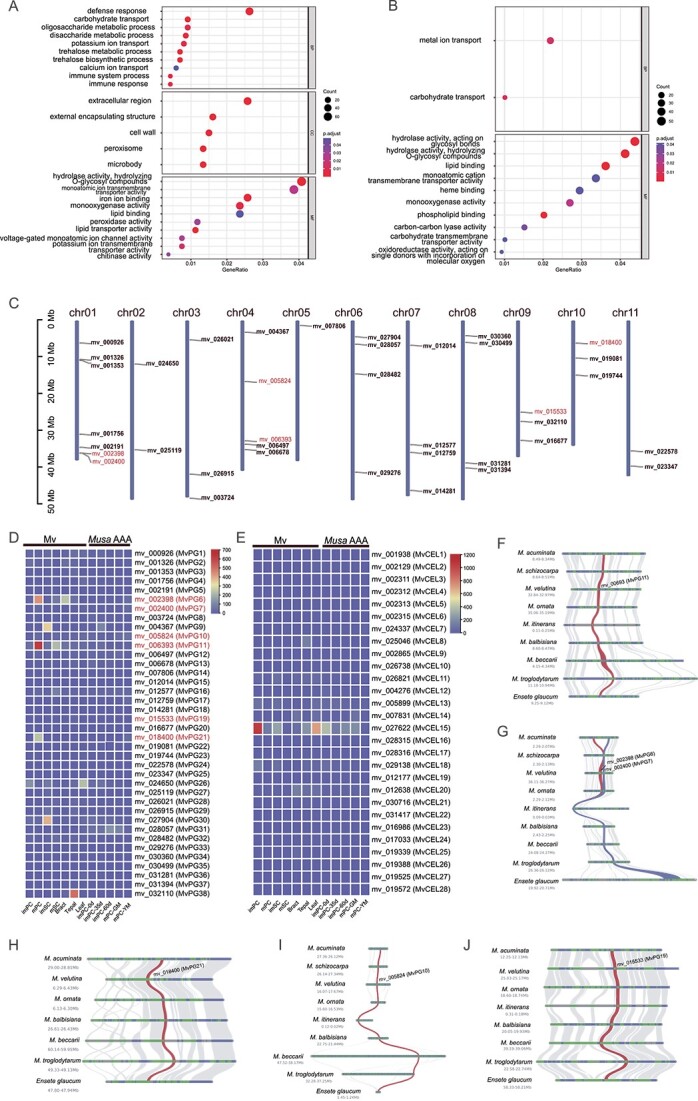
**A** and **B** GO enrichment of upregulated genes in pericarps and sarcocarps, respectively. **C** The location of PG genes on Mv chromosomes. **D** The expression levels of PG genes in different tissues of Mv and *Musa* spp. AAA. **E** The expression levels of CEL genes in different tissues of Mv and *Musa* spp. AAA. **F–J** Microsynteny of significantly upregulated PG genes across different Musaceae species. BP, biological process; CC, cellular component; imPC: immature pericarps; imPC-0d: immature pericarps from fruits just emerging from the bunch; imPC-35d: immature pericarps from 35-day-old fruits; imPC-60d: immature pericarps from 60-day-old fruits; imSC: immature sarcocarps; MF, molecular function; mPC: mature pericarps; mPC-GM: mature pericarps from green-matured fruits; mPC-YM: mature pericarps from yellow-matured fruits (6 days after ethylene treatment); mSC: mature sarcocarps.

To determine which genes were involved in the pericarp dehiscence of Mv, DEG analysis was performed. DEGs between different developmental stages of Mv were identified in pericarps (immature pericarps *vs.* mature pericarps, hereafter imPC *vs*. mPC) and sarcocarps (immature sarcocarps *vs.* mature sarcocarps, hereafter imSC *vs*. mSC). In mPC, 3070 genes were upregulated and 6871 were downregulated (Table S10, see online supplementary material). In mSC, 1967 genes were upregulated and 9925 were downregulated (Table S11, see online supplementary material). GO enrichment analysis revealed that the genes upregulated in mPC were enriched in the GO terms ‘oligosaccharide metabolic process’, ‘disaccharide metabolic process’, ‘extracellular region’, ‘cell wall’, and ‘hydrolase activity, hydrolyzing O-glycosyl compounds’ (Fig. 3A; Table S12, see online supplementary material), while in mSC, the enriched GO terms associated with the upregulated genes were ‘hydrolase activity, acting on glycosyl bonds’ and ‘hydrolase activity, hydrolyzing O-glycosyl compounds’ (Fig. 3B; Table S13, see online supplementary material). Moreover, the downregulated genes in mPC and mSC were enriched in the GO terms such as ‘response to endogenous stimulus’, ‘ribosome’, and ‘structural molecule activity’ (Fig. S15, see online supplementary material).

The PG and CEL family genes were detected by searching for protein domains using HMMER v3.3.2 [44]. In total, 38 PG and 28 CEL candidate genes were identified from the protein sequences of Mv after filtering. The PG genes were named MvPG1 to MvPG38, and CEL genes were named MvCEL1 to MvCEL28 according to their chromosomal positions (Fig. 3C). Three genes (MvPG11/mv_006393, MvPG6/mv_002398, and MvPG21/mv_18400) that were significantly upregulated according to the DEG analysis (Tables S10 and S11, see online supplementary material) exhibited higher expression levels in mPC and mSC than in imPC and imSC, bracts, tepals and leaves of Mv, while no PG genes were highly expressed throughout the development stages of the dwarf banana (*Musa* spp. AAA) (Fig. 3D; Table S14, see online supplementary material). In addition, MvPG7/mv_002400, MvPG10/mv_005824 and MvPG19/mv_015533 were also significantly upregulated (Table S10, see online supplementary material) and exhibited moderate increases in expression from imPC to mPC (Fig. 3D; Table S14, see online supplementary material). According to the GO enrichment analysis, MvPG6, MvPG7, MvPG10, MvPG11, MvPG19, and MvPG21 were involved in the molecular functions of ‘hydrolase activity, hydrolyzing O-glycosyl compounds’ and ‘hydrolase activity, acting on glycosyl bonds’ (Tables S12 and S13, see online supplementary material). The six PG genes were located on chr01, chr04, chr09, and chr11 of the Mv genome (Fig. 3C), and microsynteny analysis revealed that their adjacent regions exhibited good collinearity across different species (Fig. 3F–J). Among these PG genes, MvPG6 and MvPG7 were derived from tandem duplication (TD) (Fig. 3G; Table S9, see online supplementary material), suggesting that TD may have contributed to the pericarp dehiscence of Mv. In contrast, no CEL genes showed higher expression level in mPC and mSC than in the other tissues (Fig. 3E; Table S14, see online supplementary material).

Multiple sequence alignment revealed two variable domains (PGHG and RIK) and two relatively conserved domains (SPNTDG and GDDC) in the PG genes (Fig. S16, see online supplementary material). The protein sequence divergence of MvPG11/mv_006393 from its orthologs was low, and only one amino acid was uniquely present in MvPG11 (i.e., methionine at position 351 of the alignment). The phylogenetic tree of the PG family showed that orthologs of MvPG11 formed a monophyletic clade (Fig. S17, see online supplementary material). The Ka and Ks values between gene pairs within the PG family were calculated. The results showed that Ka and Ks were relatively high when MvPG11 was compared to its paralogs (mean Ka = 0.84, mean Ks = 1.92), but Ka and Ks were relatively low when MvPG11 was compared to its orthologs (mean Ka = 0.03, mean Ks = 0.16) (Table S15, see online supplementary material). Compared to its orthologs, MvPG11 had a mean Ka/Ks of 0.3, suggesting that this gene was under purifying or negative selection and might have a conserved function within the Musaceae and Zingiberaceae.

Previous studies have shown that PG genes can be upregulated by certain transcription factors (TFs), such as AP2/ERF, NAC, and MADS-box [45–47]. Therefore, we investigated these TFs in the Mv genome. After filtering, 444 AP2/ERF genes of Mv were obtained, among which 41 genes were upregulated in the mPC, and one gene (mv_002944) exhibited a sharply increased expression level from imPC to mPC (Fig. S18A, see online supplementary material). We identified 146 NAC genes in Mv, among which 30 genes were upregulated in the mPC, and the expression levels of three genes (mv_002857, mv_025663, and mv_030006) sharply increased from imPC to mPC (Fig. S18B, see online supplementary material). Furthermore, 73 MADS-box genes in Mv were detected, among which five genes were upregulated in the mPC, and the expression level of one gene (mv_023703) sharply increased from imPC to mPC (Fig. S18C, see online supplementary material). To determine whether AP2/ERF, NAC, and MADS-box might regulate PG genes and to facilitate future studies, we identified potential transcription factor binding sites (TFBSs) in the upstream regions of the PG genes identified above. For example, 156, 18, and 6 potential TFBSs of AP2/ERF, NAC, and MADS-box were predicted for MvPG11; for MvPG6, we predicted 13, 38, and 32 TFBSs for AP2/ERF, NAC, and MADS-box, respectively; and for MvPG21, there were 6, 94, and 60 TFBSs for AP2/ERF, NAC, and MADS-box, respectively (Table S16, see online supplementary material).

### Anthocyanin biosynthesis pathway

**Figure 4:**
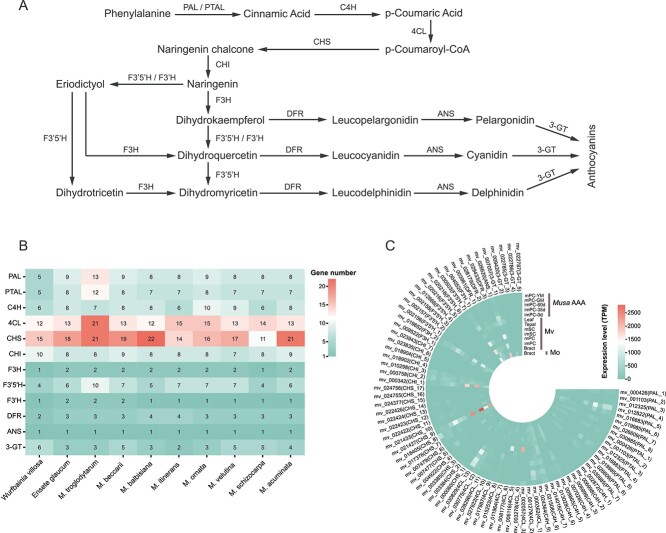
**A** Anthocyanin biosynthesis pathway. 3-GT, anthocyanidin 3-O-glucosyltransferase; 4CL, 4-coumarate CoA ligase; ANS, anthocyanidin synthase; C4H, cinnamic 4-hydroxylase; CHI, chalcone isomerase; CHS, chalcone synthase; DFR, dihydroflavonol 4-reductase; F3H, flavanone 3-hydroxylase; F3’H, flavonoid 3′-hydroxylase; F3’5’H, flavonoid 3′,5′-hydroxylase; PAL, phenylalanine ammonia-lyase; PTAL, phenylalanine/tyrosine ammonia-lyase. **B** The number of enzyme-coding genes in anthocyanin biosynthesis pathway of 10 species. The number in each cell indicates gene number. **C** Expression levels of enzyme-coding genes in the bracts of *Musa ornata* and *Musa velutina*. The expression values were standardized by the TPM method.

To facilitate horticultural breeding, we investigated the anthocyanin synthesis pathway as well as its upstream phenylpropanoid and flavonoid biosynthesis pathways (Fig. 4A). The number of genes encoding enzymes at each step was retrieved from functional annotations. The C4H gene numbers of Mo and Mv were ten and nine, respectively, which were slightly greater than those of the other species ranging from six to eight. The other structural gene numbers of Mo and Mv were similar to those of the other Musaceae species and *W. villos*a (Fig. 4B). According to the expression level analysis, several genes such as ANS_1, F3’5’H_7, F3H_2, CHS_9, 4CL_13, and PAL_4 had higher expression levels in immature pericarps (imPC-0d) than in mature pericarps (mPC-GM) of *Musa* spp. AAA (Fig. 4C; Table S17, see online supplementary material); as for Mv, ANS_1, F3’5’H_7, F3H_2, CHS_9, and 4CL_13 had higher expression levels in immature fruits (imSC and imPC) than in mature fruits (mSC and mPC) (Fig. 4C; Table S17, see online supplementary material). These findings suggested that the anthocyanin accumulation rate may be greater in immature fruits than in mature fruits, which was consistent with the findings of previous studies [48, 49]. Furthermore, ANS_1, F3’5’H_7, F3H_2, CHS_9, and 4CL_13 generally had higher expression levels than the other anthocyanin synthesis-related genes in the bracts of Mo and Mv (Fig. 4C; Table S17, see online supplementary material), suggesting that these five genes may play important roles in bract coloration. In addition, these five structural genes showed higher expression levels in imPC of Mv than in imPC of *Musa* spp. AAA (Fig. 4C; Table S17, see online supplementary material).

## Discussion

Although Mo and Mv are widely cultivated as important ornamental plants in tropical regions [24], their genomes are still lacking, which hampers the plant molecular breeding efforts aimed at enhancing desirable traits. In this study, we generated chromosome-level genomes for them. Both Mo and Mv were assembled into 11 pseudochromosomes with genome sizes of 427.85 Mb and 478.10 Mb, respectively. The contig N50, BUSCO assessment, mapping rate and LAI showed that the two genome assemblies had high continuity and completeness. Furthermore, we inferred the phylogenetic relationships and gene family expansion and contraction. DEGs in immature and mature pericarps were identified and the results showed that the significantly upregulated DEGs in mature pericarps were related primarily to saccharide metabolic processes at the cell wall or in the extracellular region. We compared the expression levels of PGs in different tissues and found that several PG genes had exceptionally high expression level in the mature pericarps. Additionally, we identified genes involved in the anthocyanin biosynthesis pathway in Mo and Mv.

Species relationships and divergence times are among the most crucial concerns for evolutionary biologists. According to our results, Mo and Mv were sister species and were closely related to *M. acuminata* and *M*. *schizocarpa*, followed by *M*. *itinerans* and *M. balbisiana*, which is largely in agreement with the findings of previous studies [18, 23]. However, conflicting phylogenetic positions were previously observed for *M*. *schizocarpa* and *M*. *itinerans* based on nuclear and plastid loci [1, 23, 50], which may suggest a complex evolutionary history within *Musa*. The Musaceae crown age (split of *Ensete* and *Musa*) was estimated to be 51.9 Mya in our study, which is largely consistent with Fu *et al.* [23] (59.19 Mya). However, the Musaceae crown age estimated by Zhou *et al.* [18] was much younger at 9.89 Mya. Because the fossil *Ensete oregonense* was recovered from the middle Eocene of western North America (43 Mya) [51], the crown age Musaceae should not be less than 43 Mya. The divergence time estimated by Zhou *et al.* [18] might be biased due to the fossils or second calibration points used.

D’Hont *et al.* [14] indicated that the *Musa* lineage had experienced three rounds of WGD events. In this study, we observed two typical ones at ~0.55 and ~ 0.9 of Ks (Fig. S11, see online supplementary material), of which the peak at 0.55 likely represents the α and β WGD events around the Cretaceous-Paleocene boundary, and the peak at 0.9 indicates the more ancient γ WGD event at approximately 100 Mya according to D’Hont *et al.* [14]. In addition, our results suggested that *Musa* species had no species-specific WGD events, which is congruent with the findings of previous studies [16, 52]. In terms of structural variation between homologous chromosomes, no inversions or rearrangements have been detected in chr04 between *M. acuminata* and *M. balbisiana* [16]. This suggests that the variations in chr04 of Mo likely emerged after its divergence from Mv. Conversely, there are no structural variations among chr05 of *M. balbisiana*, chr05 of Mo, and chr03 of Mv. This indicates that the inversions observed in chr05 of *M. acuminata* and *M. schizocarpa* probably occurred after their divergence from Mo and Mv. Genome size variations in angiosperms are determined primarily by LTRs rather than by WGD, as LTRs occupy most of the genome content [53]. According to our results, the LTR length of Mv was ~204 Mb, greater than that of Mo (~166 Mb) and *M. acuminata* (~190 Mb) (Table S5, see online supplementary material), which could explain most of the variation among the three genomes. As ancient LTRs are prone to be recognized and eliminated [54, 55], Mv (with generally older insertion times) should contain less LTRs than Mo does, which is contrary to our results but could be explained by the recent second peak at 0.5 Mya. Ancient LTRs can be eliminated by imbalanced homologous recombination and double-strand breaks [56]; thus, the lack of this removal mechanism may lead to the retainment of older LTRs in the Mv genome, which requires further investigation.

Pericarp dehiscence can facilitate seed dispersal in wild plants but result in yield loss in food crops. Although pericarp dehiscence has relatively limited impact on the value of ornamental plants, exploring its molecular mechanism could help biologists understand how this trait evolves among diverse plant groups and how it facilitates species adaptation to environment. The PG genes were shown to be essential for the development, ripening, and abscission of fig fruits [45], as well as for the pod dehiscence of *Brassica napus* [57] and *Arabidopsis thaliana* [35]. In this study, we found that several PG genes (particularly MvPG11) had higher expression levels in mature pericarps and were likely responsible for the dehiscence of Mv fruits. In contrast, no PG or CEL genes were highly expressed in the mature pericarps of *Musa* spp. AAA. According to our findings, MvPG11 was present in the Musaceae and Zingiberaceae, had quite similar amino acid sequences among different species and was under purifying or negative selection. Previous study revealed that genes under strong purifying or negative selection are functionally conserved [58]; therefore, MvPG11 may have a conserved gene function, and the dehiscence of Mv and indehiscence of other *Musa* species may be determined by the gene expression levels. Previous studies have indicated that the expression of PG genes is positively regulated by several transcription factors, such as AP2/ERF, the NAC and the MADS-box family transcription factors [45–47]. In this study, we identified potential TFBSs for PG genes and highly expressed TFs in mature pericarps; however, further experimental verification is needed. In addition, pericarp dehiscence has also been reported in the other *Musa* species, such as *M*. *schizocarpa* and some cultivars of *M. acuminata* [4, 38]. These samples should also be included in the future to explore whether the same PG genes determines pericarp dehiscence in different species and how they are regulated by TFs.

Anthocyanins are natural pigments responsible for the purple, blue, and red color in leaves, stems, flowers, fruits, and roots of plants [59]. For example, the leaves and pseudostems of *Musa* spp. AAA changed from green to purple during its development stages, but remained green when the anthocyanin synthesis-related genes (e.g., CHS, ANS, and DFR) were repressed by a MYB transcription factor [60], indicating that plant tissue colors may be determined by the expression of the anthocyanin synthesis-related genes. This study found that the expression levels of CHS_9, CHI_1, F3H_2, F3’5’H_7, and ANS_1 in the imPC of Mv were 2.56, 6.95, 1.36, 2.92, and 1.20 times more than those in imPC-0d of *Musa* spp. AAA (Fig. 4C; Table S17, see online supplementary material). The pericarp of Mv is pink during its development stages, while the pericarp of *Musa* spp. AAA is green and turns yellow-green when mature. This suggested that the differential expression of these structural genes might have led to the distinct peel colors of Mv and *Musa* spp. AAA. However, further research is needed to explore how these genes determine color formation in banana tissues.

## Materials and methods

### Plant material collection and sequencing

Fresh and young leaves of Mo and Mv were collected from the South China Botanical Garden (Guangdong, China) and subjected to genomic DNA extraction following the procedures of the Qiagen Genomic DNA Kit. Degradation of the extracted DNA was assessed by 0.75% gel electrophoresis; DNA purity was evaluated using a NanoDrop One UV–Vis spectrophotometer (Thermo Fisher Scientific, Waltham, MA, USA); and DNA concentration was measured utilizing Qubit 3.0 fluorometers (Thermo Fisher Scientific, Waltham, MA, USA). High-quality DNA was used to prepare short and long read whole-genome sequencing (WGS) libraries.

Total RNA was extracted using the TRNzol Universal RNA Extraction Kit (Tiangen, Beijing, China). RNA of Mo was extracted from leaves, tepals, and bracts. RNA of Mv was extracted from leaves, tepals, and bracts, as well as pericarps and sarcocarps at immature and mature stages.

A paired-end (2 × 150 bp) Illumina library was prepared using the TruSeq Nano DNA HT Sample Preparation Kit and subsequently sequenced using the Illumina HiSeq X Ten platform (Illumina, San Diego, CA, USA). The Nanopore library was constructed using the LSK109 Ligation Sequencing Kit (Oxford Nanopore Technologies, Oxford, UK), and sequencing was performed using a Nanopore PromethION sequencer (Oxford Nanopore Technologies, UK) at GrandOmics Co., Ltd (Wuhan, China). The Hi-C library was generated based on the method detailed in Belton *et al.* [61] with some modifications. Briefly, young and fresh leaves were fixed in nuclei isolation buffer with 2% formaldehyde. The cross-linked DNA was subsequently digested with 100 units of DpnII (New England Biolabs, USA). The digested fragments were biotinylated with biotin-14-dCTP and ligated using T4 DNA polymerase (New England Biolabs). The ligated DNA was enriched, sheared into 300- to 600-bp fragments, blunt-end repaired, and further processed. The final paired-end (2 × 150 bp) Hi-C library was sequenced on the Illumina HiSeq X Ten platform. The RNA library was constructed using the TruSeq RNA Library Preparation Kit, and RNA sequencing was carried out on the Illumina HiSeq X Ten platform with paired-end reads (2 × 150 bp).

After sequencing, fastp v0.23.3 [62] was used to remove adapters and low-quality reads with default parameters from Illumina, Hi-C and RNA reads. Porechop v0.2.4 [63] was used to remove adapters from the Nanopore long reads.

### K-mer analysis and genome assembly

Genome size was estimated using Illumina reads via Jellyfish v2.3.0 [64] and GenomeScope v1.0 [65] with a k-mer length of 21. Nanopore reads were used to assemble the genome via NextDenovo v2.5.1 [66]. Purge Haplotigs v1.1.2 [67] was utilized to identify and remove haplotypic duplications in the primary genome assemblies. Thereafter, the genome assemblies were polished using two rounds of Racon v1.5.0 [68] for Nanopore reads and hapo-G v1.3.4 [69] for Illumina reads. The polished genome assemblies were scaffolded with Hi-C reads using Juicer v1.6 [70] and 3d-dna v180922 [71] and then manually adjusted in Juicebox v1.11.08 [72]. Gaps in the genomes were filled with Nanopore reads using TGS-GapCloser v1.1.1 [73]. The gap-closed genome assemblies were further polished using Racon and hapo-G, respectively, each with two rounds. Gaps, telomeres, and centromeres were subsequently identified using quarTeT v1.1.5 [74].

### Genomic evaluation and repeat annotation

The integrity of the assembled genomes was assessed using BUSCO v5.3.2 [75] with the embryophyta_odb10.2020-09-10 database. To determine genome completeness, mapping rates were calculated by mapping Illumina reads to the genomes with BWA-MEM v0.7.17-r1188 [76] and RNA reads to genomes with HISAT2 v2.2.1 [77]. The percentage of mapped reads was subsequently determined with the ‘stats’ command in BamTools v2.5.1 [78]. Genome assembly quality was evaluated by LTR assembly index (LAI), which was calculated by using the LAI program [79]. A Hi-C interaction heatmap was generated using HiCExplorer v3 [80]. Repetitive sequence annotation was performed using the Extensive de novo TE Annotator (EDTA) v2.1.0 [81].

### Gene structure and function annotation

Genomes were masked using RepeatModeler v2.0.1 [82] and RepeatMasker v4.1.2 [83]. Gene prediction and functional annotation were performed on the soft-masked genomes using funannotate v1.8.15 [84]. Briefly, the gene prediction models were trained via the ‘funannotate train’ function based on the RNA reads. The protein-coding genes of Mo and Mv were predicted using the ‘funannotate predict’ function, which employs GeneMark-ET v3.10–5 [85], Augustus v3.5.0 [86], SNAP v2013-02-16 [87], and GlimmerHMM v3.0.1 [88]. Additionally, the tRNAs were predicted by means of tRNAscan-SE v2.0.11 [89]. In this step, protein-coding sequences of *E. glaucum*, *M. acuminata*, *M. balbisiana*, *M. itinerans*, and *M. schizocarpa* were downloaded from the Banana Genome Hub [90] as protein evidence (Table S4, see online supplementary material). Thereafter, the gene models were revised using the ‘funannotate update’ feature. InterProScan v5.62–94.0 [91] and the local version of EggNOG-mapper v2.1.11 [92] were used to identify motifs and protein domains by matching against public databases. The results of the InterProScan and EggNOG-mapper analyses were merged using the ‘funannotate annotate’ feature.

### Gene family expansion, contraction, and GO enrichment analysis

Gene orthologs and gene duplication events of Mo and Mv were identified using OrthoFinder v2.5.4 [93] by comparison with eight other species in the Musaceae and Zingiberaceae (Table S6, see online supplementary material). Based on the species tree inferred by OrthoFinder, divergence time was estimated using a penalized-likelihood method implemented in treePL v1.0 [94]. The crown age of the Zingiberales was calibrated to 83.5 million years ago (Mya) using *Spirematospermum chandlerae* [95], the oldest-known fossil of the order. The crown age of Musaceae estimated by Janssens *et al.* [50] (51.9 Mya) was used to constrain the split of *Ensete*/*Musa*. Gene family expansions or contractions were detected using CAFE v5.0 [96]. GO enrichment analysis was performed for unique gene families, as well as significantly expanded and contracted gene families using the enricher function in the R package clusterProfiler v4.8.2 [97].

### Genome synteny, duplications, and whole-genome comparisons

Genome synteny analysis was performed based on the genomes of Mo, Mv, and *M. acuminata*. The orthologs were identified and filtered with the parameters —cscore = 0.99 and —minspan = 30, and the final synteny plot was visualized using the MCscan pipeline [98] following Huang *et al.* [17]. Hi-C signals surrounding large inversions and translocations were visualized using HiCExplorer v3. Duplicated gene pairs of Mo and Mv were classified into WGD, TD, PD, TRD, and DD using the R package doubletrouble v1.0.0 [99]. *M. acuminata* was set as an outgroup in the analysis. The program WGDI v0.6.5 [100] was used to infer the polyploidization events in *M. acuminata*, Mo, and Mv. Collinear genes were identified using the ‘-icl’ option of WGDI within each genome, and Ks were calculated using the ‘-ks’ option with the Nei-Gojobori method implemented in the YN00 program in PAML v4.9h [101]. The Gaussian fitting curve parameters of each Ks peak were used to produce the Ks distribution map with the ‘-kf’ option. To model the changes in effective population size through time, the program PSMC v0.6.5-r67 [102] was used to infer the population history of Mo, Mv, and *M. acuminata* based on individual whole-genome sequences. The Illumina reads of *M. acuminata* used in this analysis were downloaded from the European Nucleotide Archive under project PRJEB35002 (Table S6, see online supplementary material). Ka/Ks were calculated for the genes of Mo and Mv using TBtools v1.120 [103] with the Nei-Gojobori model. Positively selected genes were subjected to GO enrichment analysis using the R package clusterProfiler.

### Differentially expressed gene analysis of Mv

In the present study, the dehiscent pericarp indicates the maturity of Mv fruits. RNA of Mv was sequenced for immature pericarps (imPC) and sarcocarps (imSC), as well as mature pericarps (mPC) and sarcocarps (mSC). The reads for gene exons were counted using featureCounts v2.0.6 [104]. DEGs were identified using the DEGexp function with the MARS method in the R package DEGseq v1.54.0 [105]. The DEGs between imPC and mPC as well as between imSC and mSC were selected with the criterion of absolute normalized log_2_-transformed fold-change >2 and *P*-value <0.001. GO enrichment analysis of the upregulated DEGs was performed using the enricher function in the R package clusterProfiler.

### Identification of pericarp dehiscence-related genes in mv

Based on the Hidden Markov Model (HMM) file of the polygalacturonase (PG) protein domain PF00295 from the Pfam database (https://www.ebi.ac.uk/interpro/), the PG genes were searched for within the protein sequences using HMMER v3.3.2 [44] (e-value ≤1e-5). Five protein sequences (Table S18, see online supplementary material) of the cellulase (CEL) family genes from *A. thaliana* and *Glycine max* were downloaded from the National Center for Biotechnology Information (NCBI) and aligned using MAFFT v7.508 [106]. The alignments were used to generate the HMM file, and the CEL genes were searched for within the protein sequences using HMMER (e-value ≤1e-5). Protein sequences without conserved domains or motifs were excluded. The remaining sequences were subsequently aligned using MAFFT, and sites with more than 50% gaps were removed using ClipKIT [107]. The alignment was used to construct a maximum likelihood tree in IQ-TREE v1.6.12 with 1000 ultrafast bootstraps [108]. The best-fit model (JTT + R6) was determined by ModelFinder [109] according to the BIC criterion. Nonsynonymous substitution rates (Ka), Ks and Ka/Ks were calculated using the Nei-Gojobori model in TBtools. The chromosomal location of the PG genes was illustrated with TBtools. Potential TFBSs in the promoter sequences of the PG genes were predicted using the online program JASPAR (https://jaspar.elixir.no/) with relative profile score threshold > 90% [110]. Upstream 2000 bp sequences of the PG genes were extracted for the analysis.

The AP2/ERF, NAC, and MADS-box transcription factors have been shown to upregulate the expression of PG genes that are related to fruit ripening and softening [45, 47, 111]. To explore whether these transcription factors had high expression levels in the mature pericarps, we downloaded the HMM files of AP2/ERF (PF00847), NAC (PF01849 and PF02365), and MADS-box (SRF domain PF00319 and MEF2 domain PF09047) from the Pfam database and searched the transcription factors using HMMER (e-value ≤1e-5). Besides, we downloaded the protein sequences of AP2/ERF, NAC, and MADS-box of *M. acuminata* from the PlantTFDB (http://planttfdb.gao-lab.org/), and searched for the transcription factors using blastp v2.11.0 [112] with e-value ≤1e-5, score ≥100 and coverage ≥80. The results of HMMER and blastp were combined, repeated transcription factors were removed, and conserved domains and motifs were checked.

To investigate whether the identified genes were highly expressed in mature but indehiscent pericarps, RNA from various stages of the pericarps of dwarf banana (*Musa* spp. AAA) [113] was obtained from the National Genomics Data Center (NGDC), China National Center for Bioinformation (CNCB) (Table S6, see online supplementary material). Read counts were standardized in R v4.3.1 [114] with the TPM method, which accounts for the effects of sequencing depth and gene length among different samples. A heatmap displaying gene expression levels was generated with TBtools and ChiPlot (https://www.chiplot.online/). The microsynteny of the highly expressed PG genes and adjacent regions across multiple species was visualized using the MCscan pipeline.

### Anthocyanin biosynthesis pathway

The anthocyanin biosynthesis pathway was obtained from the KEGG PATHWAY Database (https://www.kegg.jp/kegg/pathway.html). Protein sequences were functionally annotated using EggNOG-mapper. Genes encoding enzymes in the pathway were extracted from the annotations. RNA reads of Mo, Mv, and *Musa* spp. AAA were mapped to the Mv genome, and read counts were standardized using the TPM method. The gene number and expression level heatmaps were visualized using ChiPlot.

## Supplementary Material

Web_Material_uhae079

## Data Availability

All the raw sequence data were deposited in the Genome Sequence Archive in the National Genomics Data Center (NGDC), China National Center for Bioinformation (CNCB) with the accession number of CRA013014 under BioProject PRJCA020485 (https://ngdc.cncb.ac.cn/). The genome assemblies reported in this study were deposited in the Genome Warehouse in NGDC, CNCB under the accession number GWHDVGC00000000 (*M. ornata*) and GWHDVGD00000000 (*M. velutina*). In addition, the genome assemblies, protein-coding sequences, as well as genome annotations were deposited in the Science Data Bank [115] and figshare [116].
